# Assessing contamination of microalgal astaxanthin producer *Haematococcus* cultures with high-resolution melting curve analysis

**DOI:** 10.1007/s13353-016-0378-x

**Published:** 2016-11-26

**Authors:** Adam Dawidziuk, Delfina Popiel, Magda Luboinska, Michal Grzebyk, Maciej Wisniewski, Grzegorz Koczyk

**Affiliations:** 1 Functional Evolution of Biological Systems Team, Institute of Plant Genetics, Polish Academy of Sciences, Strzeszynska 34, 60-479 Poznan, Poland; 2 AlgaeLabs Ltd., Dunska 9, 54-427 Wroclaw, Poland; 3 Institute of Chemistry of Food Technology, University of Economics, Komandorska 118/120, 53-345 Wroclaw, Poland

**Keywords:** HRM, *Haematococcus pluvialis*, Diagnostics, Microalgae, Contamination

## Abstract

Due to its superior antioxidant capabilities and higher activity than other carotenoids, astaxanthin is used widely in the nutraceutical and medicine industries. The most prolific natural producer of astaxanthin is the unicellular green microalga *Haematococcus pluvialis*. The correct identification of any contaminants in *H. pluvialis* cultures is both essential and nontrivial for several reasons. Firstly, while it is possible to distinguish the main microalgal contaminant *Coelastrella* sp. (in *H. pluvialis* cultures), in practice, it is frequently a daunting and error-prone task for personnel without extensive experience in the microscopic identification of algal species. Secondly, the undetected contaminants may decrease or stop production of astaxanthin. Lastly, the presence of other contaminants such as fungi can eventually infect and destroy the whole algae collection. In this study, high-resolution melting (HRM) analysis was developed to detect microalgal and fungal contamination. The developed diagnostic procedure allowed to distinguish pure *H. pluvialis* samples from cultures contaminated with low amounts (1.25 ng/ml) of microalgal DNA and fungal DNA (2.5 ng/ml). Such discrimination is not possible with the use of microscopy observations and allows fast and efficient collection testing.

## Introduction

Astaxanthin (C40H52O4, 3,3’-dihydroxy-β,β-carotene-4,4’-dione) is a red ketocarotenoid with extraordinary antioxidant capabilities. For many years, it has been produced synthetically and used mainly in aquaculture and poultry business as a pigmentation source, with an annual turnover of over $200 million (Li et al. 2011) and a selling price of roughly $5000–6000 per kilo as of July 2012. Currently, natural astaxanthin sources gain the main attention on the market. Because of its superior antioxidant capabilities and a higher activity than that of other carotenoids, the compound is used widely in the nutraceutical and medicine industries (Guerin et al. 2003). Pertinently, natural astaxanthin is over 50 times and 20 times stronger in singlet oxygen quenching and free radical elimination, respectively, than synthetic astaxanthin (Capelli et al. 2013). This is due to the different composition of synthesized metabolite mixtures, as the natural product is predominantly a mixture of esters of a single enantiomer (3S, 3’S), while the artificial synthesis of astaxanthin results in a mixture of enantiomers in non-esterified form. The superiority of the natural source (Capelli et al. 2013) results in the final prices of natural astaxanthin being around $15,000 per kilo. Natural astaxanthin mixes are produced by various plants, bacteria, fungi, and green algae.

This unicellular green microalga seems to be able to accumulate the highest levels of astaxanthin among all natural sources amenable to mass culturing. It accumulates astaxanthin up to 5% of its cell dry weight. The best results for commercial astaxanthin production are achieved by using closed system photobioreactors, because of the smaller water losses and lower risk of contamination in comparison to open systems (Lorenz and Cysewski 2000).

**Fig. 1:**
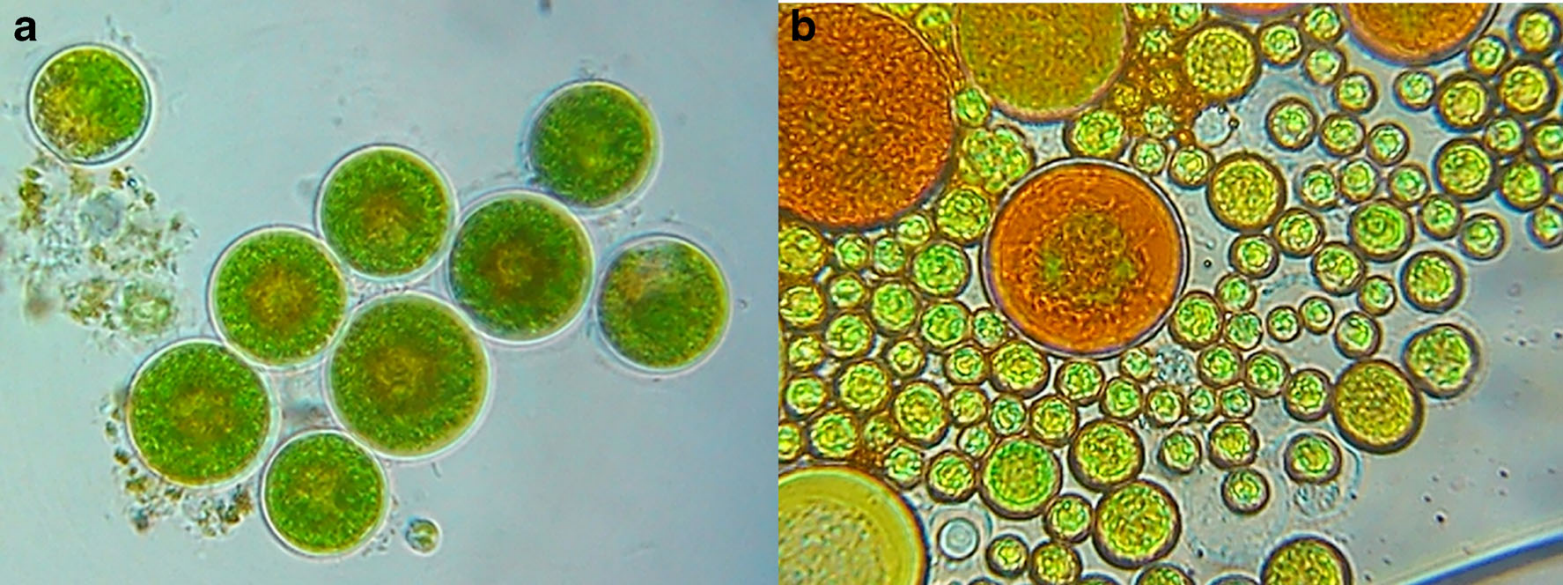
**a** *Haematococcus pluvialis* cells. **b** Both *H. pluvialis* (bigger) and *Coelastrella* sp. (smaller) cells

It is possible to distinguish the contaminations of *Haematococcus* cultures, with other common microalgae, using a combination of morphological differences on the microscopic level. However, in a high-throughput setting, where the presence/concentration of contaminants is emphasized over their nature, this approach is both time-consuming and error-prone. In the authors’ collective experience, the identification of a common contaminant (*Coelastrella* sp.; see also Fig. 1a, b for a visual comparison with *Haematococcus* culture) has proven difficult for laboratory personnel without extensive experience in microscopic techniques. For this reason, it was advantageous to develop a quick molecular diagnostic technique yielding a binary outcome (distinguishing between contaminated and pure samples) for the preanalysis of samples.

For the task at hand, the use of the simplest methods, such as polymerase chain reaction (PCR), tends not to provide reliable results due to the sample being composed of several species. The basic amplification schemes can indicate only the dominant organism and the detection of multiple contaminants with separate species-specific primers would require a large number of marker combinations or complex multiplexing schemes. Our proposed resolution is to, instead, use the highly efficient and sensitive high-resolution melting (HRM) analysis (Penna and Galluzzi 2013) with a reference, pure culture. The HRM assessment constitutes a homogeneous and efficient diagnostic approach and is frequently utilized for single nucleotide polymorphism (SNP) genotyping, mutation probing in DNA samples (Granados-Cifuentes and Rodriguez-Lanetty 2011). The technique characterizes nucleic acid samples based on detecting small differences in melting (dissociation) curves of different nucleic acid duplexes formed by distinct amplification products. The samples are, thus, discriminated according to, among others, sequence length, base pair content, and strand complementarity, all of which influence the melting process. The approach adopted mostly in medical sciences (Fassina et al. 2009) can be readily exploited in a microbiological context in order to assess the presence of contaminants that are hard to distinguish with traditional methods.

The present study concerns the development and testing of an optimized HRM procedure able to efficiently distinguish pure and contaminated *Haematococcus* sp. cultures used in astaxanthin production.

## Materials and methods

### Microalgae strains and culture conditions

The tested samples described in the experiment belong to two strains of *H. pluvialis*: *H. pluvialis* G 1002 Flotow obtained from the Culture Collection of Algae of Charles University Department of Botany and *H. pluvialis HPM* obtained by the company AlgaeLabs Ltd. (Table 1). The stock cultures were grown in standard 3N-BBM medium containing: 0.75 g/l^−1^ NaNO_3_, 0.025 g/l^−1^ CaCl_2_xH_2_O, 0.075 g/l^−1^ MgSO_4_x7H_2_O, 0.075 g/l^−1^ K_2_HPO_4_, 0.175 g/l^−1^ KH_2_PO_4_, 0.025 g/l^−1^ NaCl, 0.05 g/L^−1^ EDTA 0.031 g/l^−1^ KOH, 0.00498 g/l^−1^ FeSO_4_x7H_2_O, 0.01142 g/l^−1^ H_3_BO_3_, 0.00882 g/l^−1^ ZnSO_4_x7H_2_0, 0.00144 g/l^−1^ MnClx4H_2_O, 0.00071 g/l^−1^ MoO_3_, 0.00157 g/l^−1^ CuSO_4_x5H_2_O, and 0.0049 g/l^−1^ Co(NO_3_)_2_x6H_2_O (Sigma-Aldrich, St. Louis, MO, USA). Cultures were carried out in 250-ml narrow-neck Erlenmeyer flasks covered with cotton corks, filled with 100 ml of medium with the addition of 20% of inoculate. Erlenmeyer flasks with medium were inoculated with 2.8 × 10^5^ cells of *Haematococcus*/ml culture. Growth of microalgae were carried out in a laboratory shaker (WL-2000, JW Electronic, Warsaw, Poland) with continuous operation, at 70 rpm. The green cells were cultured under continuous illumination of ca. 90 μmol m^−2^ s^−1^ from two fluorescent tubes, at 25 °C ± 1 °C, without additional aeration. No additional source of CO_2_ was used. Every 2 days, cultures were observed using a fluorescent optical microscope (Opta-Tech MN800 FL, Warsaw, Poland) at 400× magnification.

**Table 1:** Source of microalgae samples

Sample name	Sample source	Culture contamination
Alg1	*HPM*: control sample	−
Alg2	*HPM*: sample from rotary shaker	−
Alg3	*HPM*: sample from photobioreactor	−
Alg4	*HPM*: sample from plate culture	−
Alg5	G 1002 Flotow: sample from the Culture Collection of Algae of Charles University Department of Botany; stored in AlgaeLabs Ltd.	+
Alg6	*HPM*: sample producing low amounts of astaxanthin	+
Alg7	*HPM*: second sample from rotary shaker (taken 30 days after first sample)	−
Alg8	*HPM*: third sample from rotary shaker (taken 30 days after first sample)	−
Alg9	*HPM*: second sample producing low amounts of astaxanthin	+
Alg10	*HPM*: third sample producing low amounts of astaxanthin	+
Alg11	G 1002 Flotow: second sample from the Culture Collection of Algae of Charles University Department of Botany; stored in AlgaeLabs Ltd.	+
Alg12	G 1002 Flotow: third sample from the Culture Collection of Algae of Charles University Department of Botany; stored in AlgaeLabs Ltd.	+

### Packaging transport of the samples

Samples were taken directly from narrow-neck Erlenmeyer flasks under a laminar flow chamber and transferred to sterile test PP tubes of 15 ml capacity. All samples from AlgaeLabs Ltd. were described and labeled. The tubes were placed in a Styrofoam box, to provide additional measures protecting it from overheating and mechanical shock. The samples were delivered to the Institute of Plant Genetics of the Polish Academy of Sciences no more than 48 h later.

### DNA extraction

#### Reference fungal cultures

The reference fungal species (*Fusarium graminearum* Schwabe), although not a common contaminant of algal cultures, was selected as one of the most common plant pathogens in Poland, one producing high quantities of airborne spores. It was selected to test the whether presence of an uncommon contaminant (a distant fungal species) would be picked up by the HRM technique at different contamination levels. Fungal mycelium (reference strains (76L; Dawidziuk et al. 2014) used to analyze the sensitivity of the HRM test) used for DNA extraction was grown in Czapek-Dox broth (Sigma Aldrich, St. Louis, MO, USA) with yeast extract (Oxoid, Thermo Fisher Scientific, Inc., Waltham, MA, USA) and streptomycin sulfate (50 mg l^−1^, AppliChem, Darmstadt, Germany). Mycelium was collected on filter paper in a Büchner funnel and freeze-dried. Total DNA was extracted using the cetyltrimethylammonium bromide (CTAB) method (Doohan et al. 1998).

#### Algae samples

Samples for DNA extraction were grown in NIES-C medium. Algae thalli was rinsed in the sterile water, blot dried, and ground to a fine powder in liquid nitrogen. DNA was obtained by using a modified DNase kit (Qiagen, Hilden, Germany) methodology. The quality of DNA was assessed by the NanoDrop 2000 UV–vis spectrophotometer (Thermo Fisher Scientific, Inc., Waltham, MA, USA) and via the Experion Automated Electrophoresis System (Bio-Rad, Hercules, CA, USA).

### Primer design

Primers were designed on the basis of the reference *Haematococcus pluvialis* 18S ribosomal RNA gene sequence (KF644445) from NCBI/GenBank release version 194 (Benson et al. 2013) (Table 2). Primer sequences were screened against propensity for homodimer (Gibbs’ free energy of no less than −4 kcal/M) and heterodimer (Gibbs’ free energy of no less than −5 kcal/M) formation. The melting temperature and propensities were judged on the basis of nearest-neighbor energy approximations carried out by the IDT OligoAnalyzer program (https://eu.idtdna.com/calc/analyzer).

**Table 2:** The sequences of primers used in the experiment

Gene targeted	Primer name	Sequences (5′-3′)	Estimated product length (base pairs)
18S ribosomal RNA gene	18S rRNA_am_fA1	AAACGGCTACCACATCCAA	115
	18S rRNA_am_rA1	CTCATTCCAATTACCAGA	
	18S rRNA_am_fA2	AATCGCCTAGCTCAACCA	121
	18S rRNA_am_rA2	ATTGTTCTCATTCCAATTACCA	
Internal transcribed spacer	ITS 4	TCCTCCGCTTATTGATATGC	760 (*Haematococcus* sp.) 680 (*Coelastrella* sp.)
	ITS 5	GGAAGTAAAAGTCGTAACAAGG	

### PCR amplification

The PCR reaction was carried out in a 25-μl reaction mixture containing the following: 1 μl of DNA (50 ng/μl^−1^), 12.5 μl PCR buffer (50 mmol/l^−1^ KCl, 1.5 mmol/l^−1^ MgCl_2_, 10 mmol/l^−1^ Tris–HCl, pH 8.8, 0.1% TritonX-100), 1U polymerase (Sigma Aldrich, St. Louis, MO, USA), 10 mmol/l^−1^ dNTP (Invitrogen, Carlsbad, CA, USA), 0.5 μl 100 mmol/l^−1^ of each primer, and 11.5 μl H_2_O. Amplifications were performed in a C1000 Touch™ Thermal Cycler (Bio-Rad, Hercules, CA, USA) under the following conditions: initial denaturation for 5 min at 94 °C, 35 cycles of 45 s at 94 °C, 45 s at 53–56 °C, 1 min at 72 °C, and a final extension for 10 min at 72 °C. Amplification products were separated on a 1.5% agarose gel (Invitrogen, Carlsbad, CA, USA) in 1 × TBE buffer (0.178 mol/l^−1^ Tris-borate, 0.178 mol/l^−1^ boric acid, 0.004 mmol/l^−1^ EDTA) and stained with ethidium bromide. The 10-μl PCR products were combined with 2 μl of loading buffer (0.25% bromophenol blue, 30% glycerol). A 100-bp DNA Ladder Plus (Fermentas, Thermo Fisher Scientific, Inc., Waltham, MA, USA) was used as a size standard. PCR products were electrophoresed at 3 Vcm^−1^ for about 2 h, visualized under UV light, and photographed (Gel DOC EZ Imager, Bio-Rad, Hercules, CA, USA).

### Sequencing

The primary analysis of algae contaminants and fungal reference strains was performed using ITS 1/2 marker (White et al. 1990) (Table 1). The 3-μl PCR products were purified with exonuclease I and shrimp alkaline phosphatase according to Chelkowski et al. (2002). Sequencing reactions were prepared using the ABI Prism BigDye Terminator Cycle Sequencing Ready Reaction Kit in 5-μl volumes (Applied Biosystems, Foster City, CA, USA). DNA sequencing was performed on an ABI PRISM3100 Genetic Analyzer (Applied Biosystems, Foster City, CA, USA).

The sequences were edited and assembled using Chromasv.1.43 (Applied Biosystems, Foster City, CA, USA). Both CLUSTAL W (Thompson et al. 1994) and MUSCLE (Edgar 2004) were used to align the sequences initially; the resulting alignments were merged and refined manually. All positions containing gaps and missing data were eliminated from the dataset.

### HRM curve analysis

The HRM curve analysis was conducted using the SensiFAST HRM Kit (Bioline Reagents Ltd., London, UK). The total reaction volume was 20 μl: 10 μl 2 × SensiFAST HRM mix, 4 μl DNA (<35 ng), 0.8 μl each primer (10 μM), and 5.2 μl nuclease-free water. The HRM curve was determined using a CFX96 Touch™ Real-Time PCR Detection System (Bio-Rad, Hercules, CA, USA). The reaction was carried out using the following protocol: initial denaturation at 95 °C for 2 min, followed by 40 cycles at 95 °C for 5 s, 60 °C for 10 s, and 72 °C for 10 s. The melting curve analysis (from 70 to 95 °C) was used to confirm primer pairs specificity and for the main analysis. A pure *H. pluvialis* sample (Alg1) was used as a reference control. In order to assess the efficiency of the reaction, a standard curve analysis was carried out. To analyze the sensitivity of the reaction, a control sample (containing 50 ng/μl of *H. pluvialis* DNA; approximately 3 × 10^5^ cells/ml) was contaminated with series of algae (*Coelastrella* sp.) and fungal (*Fusarium graminearum*) DNA in series of dilutions. For this, series of dilutions (10-, 100-, and 1000-fold) were prepared on the reference cultures of *Coelastrella* sp. (DNA content of 125 ng/ml; approximately 4 × 10^4^ cells/ml) and *F. graminearum* (250 ng/ml).

## Results

### Identification of algae and fungal cultures

**Fig. 2:**
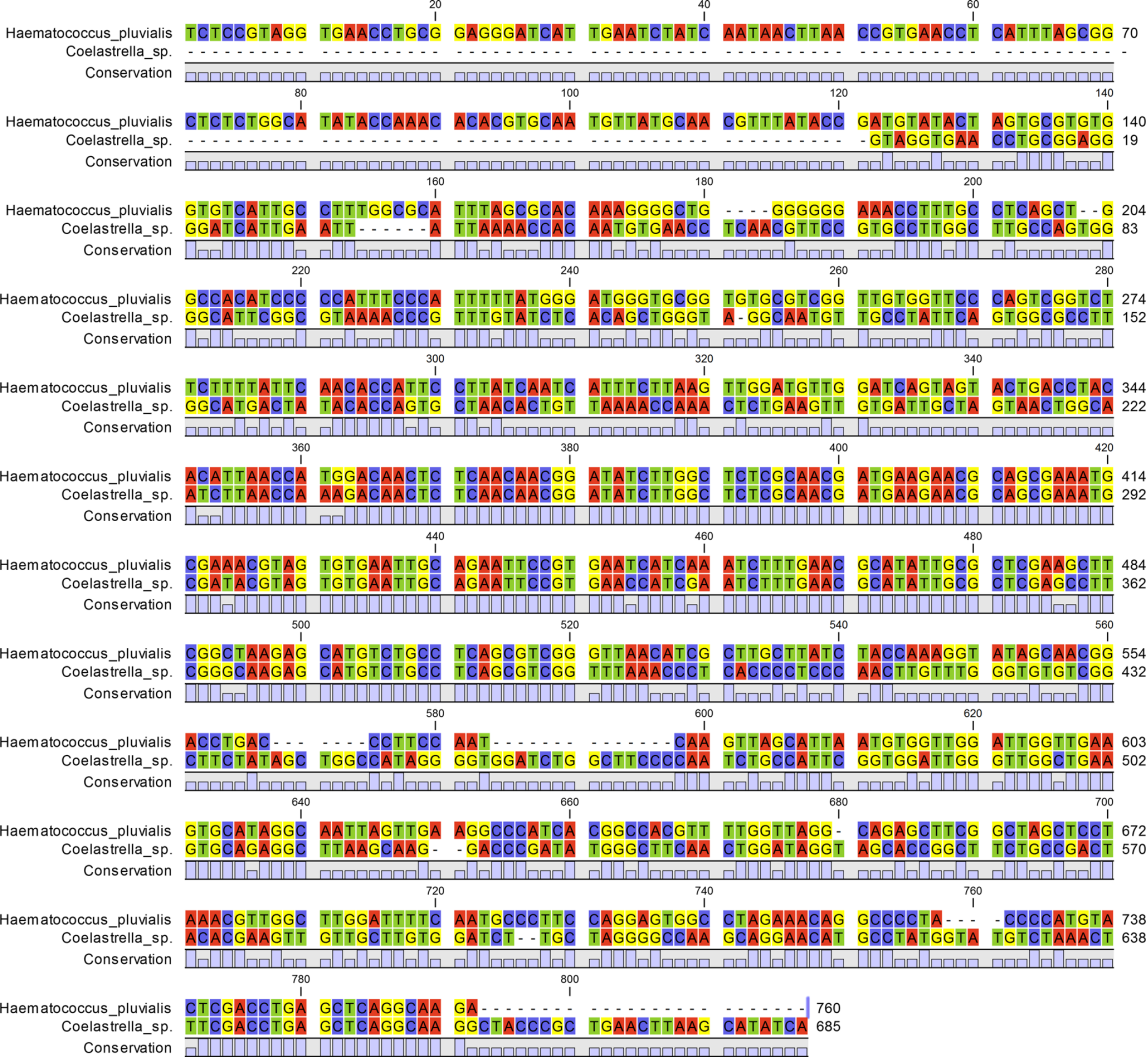
Alignment of *Haematococcus pluvialis* and *Coelastrella* sp. ITS fragments

Algae contaminants and fungal reference strains were identified using sequencing of ITS 1/2 marker. After PCR and sequencing, the obtained fragments (ca. 700 bp long) allowed to verify control samples as a *H. pluvialis* and the main algal contaminant as a *Coelastrella* sp. (Fig. 2). The Sanger sequencing of amplicons was possible only in the samples containing pure control strain of *H. pluvialis* and samples very highly contaminated with *Coelastrella* sp. (the *H. pluvialis* was not present in those samples, e.g., Alg 5). Sensitive analysis of samples contaminated with low amounts of co-inoculated microalga or fungal mycelium was not possible by sequencing.

### PCR analysis

**Fig. 3:**
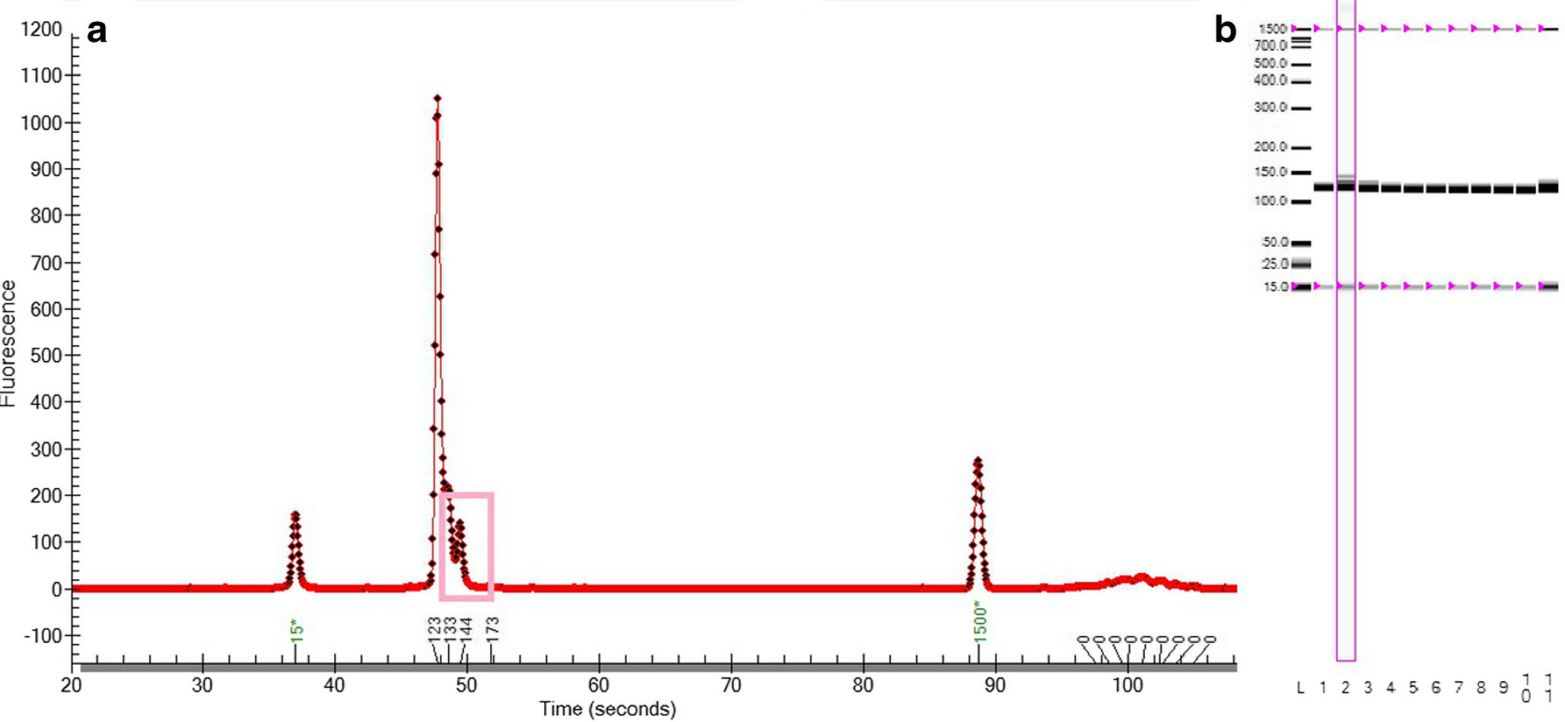
Analysis of algae samples after 18S rRNA amplification **a** Electrophoretogram view of sample highly contaminated with *Coelastrella* sp. **b** Preprocessed gel view. Samples: *1* control *Haematococcus pluvialis* sample; *2* sample contaminated with 12.5 ng/μl of *Coelastrella* sp. DNA; *3* sample contaminated with 1.25 ng/μl of *Coelastrella* sp. DNA; *4* sample contaminated with 125 pg/μl of *Coelastrella* sp. DNA; *5* sample contaminated with 10 pg/μl of *Coelastrella* sp. DNA; *6* sample contaminated with 1 pg/μl of *Coelastrella* sp. DNA; *7* sample contaminated with 25 ng/μl of *Fusarium graminearum* sp. DNA; *8* sample contaminated with 2.5 ng/μl of *F. graminearum* DNA; *9* sample contaminated with 250 pg/μl of *F. graminearum* DNA; *10* sample contaminated with 25 pg/μl of *F. graminearum* DNA; *11* sample contaminated with 2.5 pg/μl of *F. graminearum* DNA; *L* ladder. Figure printed from Experion Automated Electrophoresis System software (Bio-Rad, Hercules, CA, USA)

The designed 18S rRNA primers amplified efficiently both in the control (not contaminated) samples and in the potentially contaminated cultures. The total concentration of DNA in the amplified samples ranged between 13 ng/μl in culture contaminated with fungal DNA and 30.1 ng/μl in the control sample. As remarked, the manual analysis of PCR products by electrophoretogram and preprocessed gel view (Experion Automated Electrophoresis System, Bio-Rad, Hercules, CA, USA) allowed us to detect only contamination with a very high concentration of *Coelastrella* sp. (12.5 ng/ml) (Fig. 3). The cultures contaminated by fungal DNA (artificially added exemplary *Fusarium graminearum* strain) could not be visually distinguished from pure samples.

### Discrimination of algae cultures contamination by HRM

The designed primers amplified an approximately 120-bp-long fragment of the 18S rRNA. The standard curve analysis performed with the use of the control *H. pluvialis* sample confirmed the high efficiency of the reaction (18S rRNA_am_fA1/18S rRNA_am_rA1 pair: E = 100.2%, slope = −3.316; 18S RNA_am_fA2/18S RNA_am_rA2 pair: E = 99.9%, slope = −3.324). These values are well within the desired parameters, as the recommended efficiency of the HRM analysis should be between 98 and 102% and the slope value should fall in the range of −3.6 ≥ slope ≥ −3.3 (Garritano et al. 2009).

**Fig. 4:**
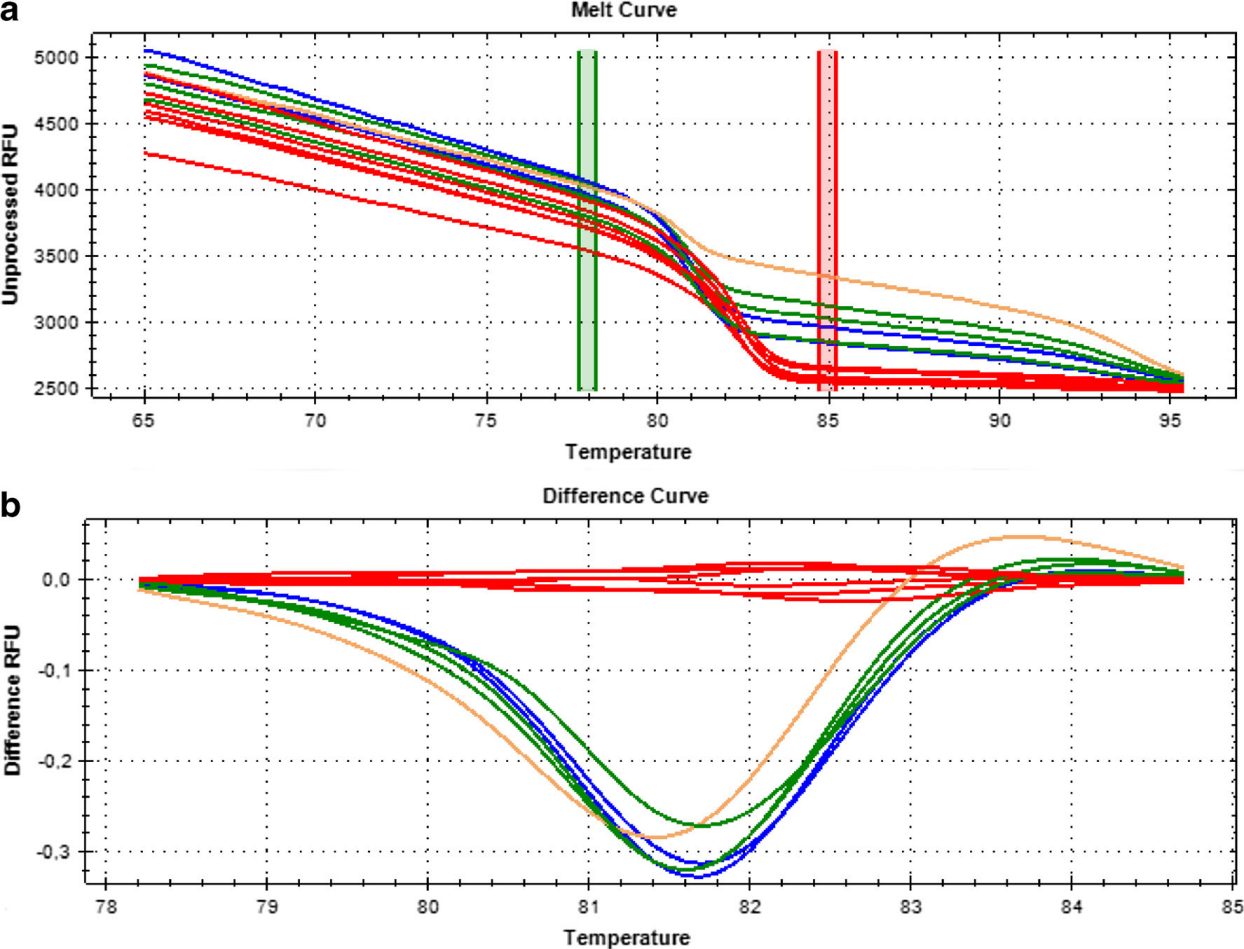
HRM analysis of algae samples using primers 18S rRNA_am_fA1 and 18S rRNA_am_rA1. **a** Melting curves profiles. **b** Difference profiles. The *red lines* indicate clustering of uncontaminated *H. pluvialis* samples (Alg1, Alg2, Alg3, Alg4, Alg7, and Alg8). The *blue*, *green*, and *brown lines* indicate cultures contaminated with *Coelastrella* sp. Figure parts printed from Precision Melt Analysis™ software (Bio-Rad, Hercules, CA, USA)

The HRM analysis on the amplified product allowed clear discrimination of pure and contaminated *H. pluvialis* cultures (see Fig. 4; the results were confirmed by the second primer set). The unsupervised clustering of melting curves on both tested primer pairs yielded correct groupings of pure cultures with the reference sample (Alg1) in Cluster 1 (samples Alg1, Alg2, Alg3, Alg4, Alg7, and Alg8). The best clustering results were obtained with the pre-melt and post-melt regions of, respectively, 77.7–78.2 °C and 84.7–85.2 °C. The temperature shift bar height, the melt curve shape sensitivity for cluster detection, and the T_m_ difference threshold were set to, respectively, 0.20, 50, and 0.15.

### Limits of sensitivity for the diagnostic markers

**Fig. 5:**
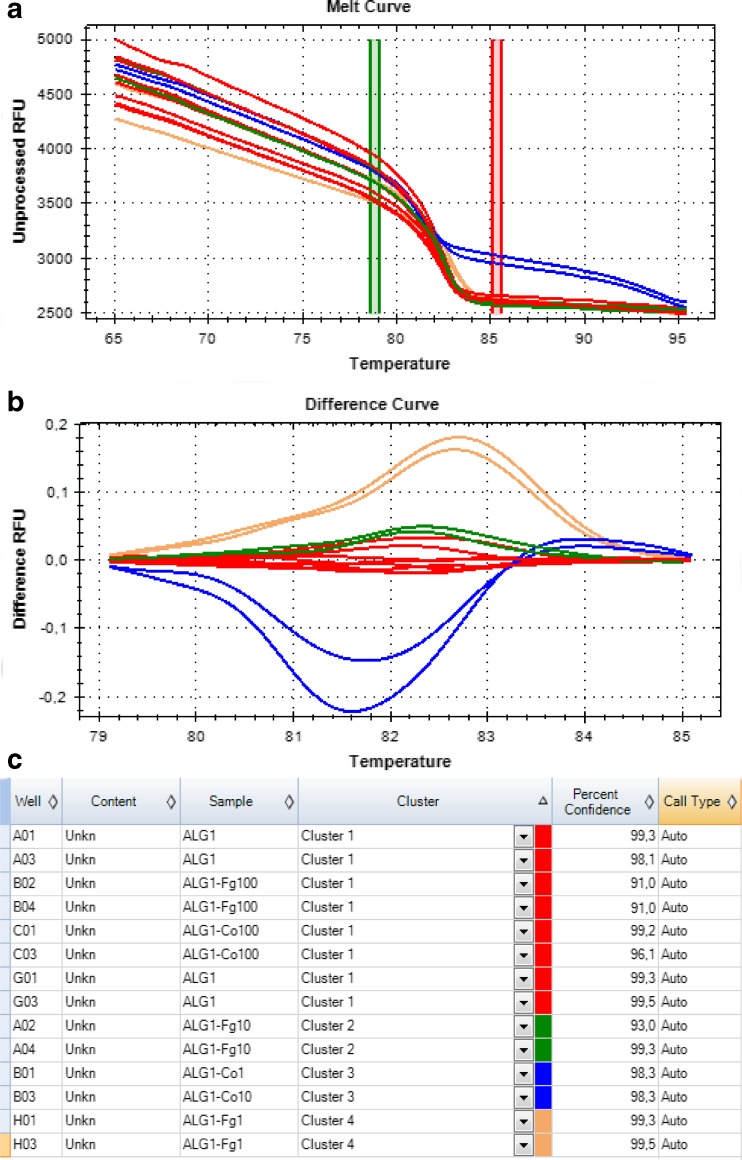
Sensitivity of the HRM analysis with the use of 18S rRNA_am_fA1 and 18S rRNA_am_rA1 primers. **a** Melting curves profiles. **b** Difference profiles. **c** Clustering of the results. Samples: *ALG1* control *Haematococcus pluvialis* sample; *ALG1-Co1* sample contaminated with 12.5 ng/μl of *Coelastrella* sp. DNA; *ALG1-Co10* sample contaminated with 1.25 ng/μl of *Coelastrella* sp. DNA; *ALG1-Co100* sample contaminated with 125 pg/μl of *Coelastrella* sp. DNA; *ALG1-Co10* sample contaminated with 10 pg/μl of *Coelastrella* sp. DNA; *ALG1-Fg1* sample contaminated with 25 ng/μl of *Fusarium graminearum* sp. DNA; *ALG1-Fg10* sample contaminated with 2.5 ng/μl of *F. graminearum* DNA; *ALG1-Fg100* sample contaminated with 250 pg/μl of *F. graminearum*. Figure parts printed from Precision Melt Analysis™ software (Bio-Rad, Hercules, CA, USA)

The designed diagnostic markers used in the HRM analysis allowed us to distinguish pure *H. pluvialis* samples from cultures contaminated with algae and artificially added fungal DNA. The contamination with *Coelastrella* sp. was recognized at contaminant DNA concentrations of both 12.5 ng/ml (approximately 4 × 10^3^ cells/ml) and 1.25 ng/ml (approximately 4 × 10^2^ cells/ml). Comparable sensitivities were observed for fungi-contaminated samples using concentrations of 25 ng/ml and 2.5 ng/ml of fungal DNA (*F. graminearum*). Contamination was not detected in samples of lower DNA content (below ca. 1 ng/ml) (Fig. 5).

## Discussion

As algae cultivation continues to grow worldwide, the prevalence of undesirable organisms in algal cultures will be a more widely reported problem. Contaminations become a potential limiting factor for production, as many algal operations scale up for mass algae production. The cultures of astaxanthin-producing *Haematococcus* sp. are potentially vulnerable to both fungal parasites and zooplanktonic predators (e.g., amoebas, ciliates and rotifers), as well as other microalgae and cyanobacteria, all resulting in reduced biomass yield and quality, and sometimes loss of culture all together. The most known and devastating contaminant of *H. pluvialis* cultures is a parasitic blastoclad fungus identified as *Paraphysoderma sedebokerensis* (Hoffman et al. 2008; Gutman et al. 2009). However, the infection by this pathogen is fairly easy to notice because, during the infection process, healthy green culture turns dark brown, accompanied by the formation of large clumps consisting of cells and cell debris (Hoffman et al. 2008). The more problematic, though less damaging, is contamination with other microalgae, which is largely asymptomatic. There is virtually no possibility to distinguish contaminant from pure culture under the microscope and specific staining can be used only in the case of fungal contamination (Damiani et al. 2006). Similarly, flow cytometry techniques often used to detect contaminated cultures (Day et al. 2012) are inadequate in the case of algal contamination.

To combat this flaw, molecular diagnostic methods that have been developed for ecological studies can offer alternatives for the identification and detection of parasites in algal culture. Primary molecular identification can be carried out by Sanger sequencing of isolated DNA templates. On the basis of previously sequenced DNA fragments, amplification of or oligonucleotide hybridization to specific target regions can be employed for the specific detection of a particular contaminating species. One of the most potent and most sensitive detection methods employs qPCR (Botes et al. 2013) and its modifications in the form of HRM curve resolution. This method, employed in our experiments to detect contaminated *H. pluvialis* cultures, proves to be efficient, fast, and cost-effective. In commercial culture samples (AlgaeLabs Ltd., Wroclaw, Poland), tested in the Institute of Plant Genesis, the main detected contaminant was identified as *Coelastrella* sp. (on the basis of the ITS 1/2 fragment sequencing). Using two separate sets of primers designed to amplify 18S ribosomal RNA gene, the HRM assay allowed the detection of both reference fungal and algal contaminants. From 12 commercial *H. pluvialis* cultures, six were confirmed as contaminated by *Coelastrella* sp.

In summary, the developed method allows for the detection of low concentrations of *Coelastrella* (1.25 ng/μl, approximately 4 × 10^2^ cells/ml) and reference fungal DNA (2.5 ng/μl) in the tested samples. Importantly, due to the high overall conservation of 18S rRNA sequences in eukaryotic organisms, the applied markers are very likely able to detect not only *Coelastrella* sp. and *Fusarium* sp. in the microalgae cultures, but also other contaminating organisms in a fast and cost-effective manner. The entire process, from DNA isolation to ending analysis, takes about 5 h (depending on the number of samples) and allows the analysis of multiple samples in a short period of time without sacrificing the quality of the results. The obtained results confirm both the applicability and efficiency of HRM-based diagnostics for applied use in the commercial culturing of biosynthetic microalgae.
